# The aorta after coarctation repair – effects of calibre and curvature on arterial haemodynamics

**DOI:** 10.1186/s12968-019-0534-7

**Published:** 2019-04-11

**Authors:** Michael A. Quail, Patrick Segers, Jennifer A. Steeden, Vivek Muthurangu

**Affiliations:** 1 Centre for Translational Cardiovascular Imaging, Institute of Cardiovascular Science, University College London and Great Ormond Street Hospital for Children, London, WC1N 3JH UK; 2 IBiTech-bioMMeda, iMinds Medical IT, Ghent University, De Pintelaan 185, 9000 Ghent, Belgium

**Keywords:** Congenital heart disease, Coarctation of the aorta, Hypertension, Cardiac magnetic resonance imaging, Hemodynamics

## Abstract

**Background:**

Aortic shape has been proposed as an important determinant of adverse haemodynamics following coarctation repair. However, previous studies have not demonstrated a consistent relationship between shape and vascular load. In this study, 3D aortic shape was evaluated using principal component analysis (PCA), allowing investigation of the relationship between 3D shape and haemodynamics.

**Methods:**

Sixty subjects (38 male, 25.0 ± 7.8 years) with repaired coarctation were recruited. Central aortic haemodynamics including wave intensity analysis were measured noninvasively using a combination of blood pressure and phase contrast cardiovascular magnetic resonance (CMR). 3D curvature and radius data were derived from CMR angiograms. PCA was separately performed on 3D radius and curvature data to assess the role of arch geometry on haemodynamics. Clinical findings were corroborated using 1D vascular models.

**Results:**

There were no independent associations between 3D curvature and any hemodynamic parameters. However, the magnitude of the backwards compression wave was related to the 1st (r = − 0.36, *p* = 0.005), 3rd (r = 0.27, *p* = 0.036) and 4th (r = − 0.31, *p* = 0.017) principle components of radius. The 4th principle componentof radius also correlated with central aortic systolic pressure. These aortas had larger aortic roots, more transverse arch hypoplasia and narrower aortic isthmuses.

**Conclusions:**

There are major modes of variation in 3D aortic shape after coarctation repair witha modest association between variation in aortic radius and pathological wave reflections, but not with 3D curvature. Taken together, these data suggest that shape is not the major determinant of vascular load following coarctation repair, and calibre is more important than curvature.

**Electronic supplementary material:**

The online version of this article (10.1186/s12968-019-0534-7) contains supplementary material, which is available to authorized users.

## Introduction

Operative repair of aortic coarctation in childhood is highly successful and associated with low mortality. However, blood pressure remains elevated, even in patients with no recoarctation [1, 2]. This suggests that post repair, patients have an abnormal vascular phenotype.

Several studies have shown that elevated aortic stiffness explains much of this hypertensive phenotype [3, 4]. More recently, increased backwards wave reflections have also been identified as a cause of increased load and higher left ventricular (LV) mass [1]. 

Another aspect of vascular phenotype that is often implicated in determining abnormal load in these patients is aortic shape. Acute arch angulation (the gothic arch) has been proposed as an important determinant of increased load [4, 5]. In particular, the gothic arch has been associated with exercise hypertension and abnormal flow profiles that are suggestive of increased wave reflections [4, 5]. However, other studies have demonstrated no relationship between shape and exercise hemodynamics [6]. Furthermore, simple evaluation of flow profiles may not accurately quantify reflected waves.

One reason for these inconsistencies may be inadequate description of the 3D aortic shape. The shape of the aorta is complex, including continuous variation of both curvature and radius along its length. Few studies have attempted to capture this complexity and those that do, have not separated radius and curvature [7, 8]. 

One method of evaluating 3D aortic anatomy is contrast enhanced cardiovascular magnetic resonance (CMR) angiography, [9] which can be processed to extract curvature (i.e. the centreline) and radius information. This data can then be further evaluated using principal component analysis (PCA) to quantify the major modes of variation within a population [10]. CMR also provides accurate measures of LV structure and aortic hemodynamics, allowing the effect of aortic shape to be determined. Importantly, this includes wave reflections that have been proposed as the mechanism by which gothic arches increase vascular load [4, 5]. 

The aims of this retrospective study were: i) to characterize the major modes of variation in aortic shape components (curvature and radius) in patients post coarctation repair, ii) evaluate any associations between aortic shape components and aortic hemodynamics or cardiac structure, and iii) validate clinical observations using 1D vascular models.

## Materials and methods

### Subjects

Sixty patients with coarctation of the aorta repaired in childhood referred for surveillance CMR were identified. Exclusion criteria were: (i) coarctation associated with major or unrepaired congenital heart disease (exception non-stenotic bicuspid aortic valve or repaired ventricular/atrial septal defects); (ii) coarctation stents; (iii) echocardiographic or CMR evidence of recoarctation (diastolic flow continuation in descending aorta or coarctation index < 0.7); (v) aortic stenosis; (vi) irregular heart rates; (vii) CMR-incompatible implants; and (Viii) pregnancy. Noninvasive hemodynamic data for 44 of the study patients has been previously reported [1]. 

### CMR protocol

All imaging was performed on a 1.5 T CMR scanner (Avanto, Siemens Healthineers, Erlangen, Germany) using two spine coils and one body-matrix coil. A vector electrocardiographic system was used for cardiac gating. Brachial systolic (p-SBP), diastolic (DBP) and mean (MBP) blood pressures were measured during the CMR scan using automated oscillometric sphygmomanometry (Datex Ohmeda, GE Healthcare). Small-adult, adult and large-adult cuff sizes were chosen according to subject arm circumference and all measurements were taken from the patient’s right arm. Blood pressures were assessed at least 15 min into the scan protocol (at the time of flow imaging) to ensure acclimatization to the supine position.

### Aortic shape assessment

Aortic arch anatomy was assessed in patients using contrast enhanced CMR angiography as previously described [11]. Briefly, images were acquired with a 3D spoiled gradient echo sequence with an isotropic resolution of 1.2 × 1.2 × 1.2 mm. Gadolinium (0.2mMol/kg, Dotarem, Guerbet, Villepinte, France) was injected into a peripheral vein and the CMR angiographic sequence was initiated when contrast reached the aorta. Two consecutive angiograms were acquired in separate 15–20s breath holds. The early angiogram with higher aortic contrast was used for subsequent post processing.

**Fig. 1:**
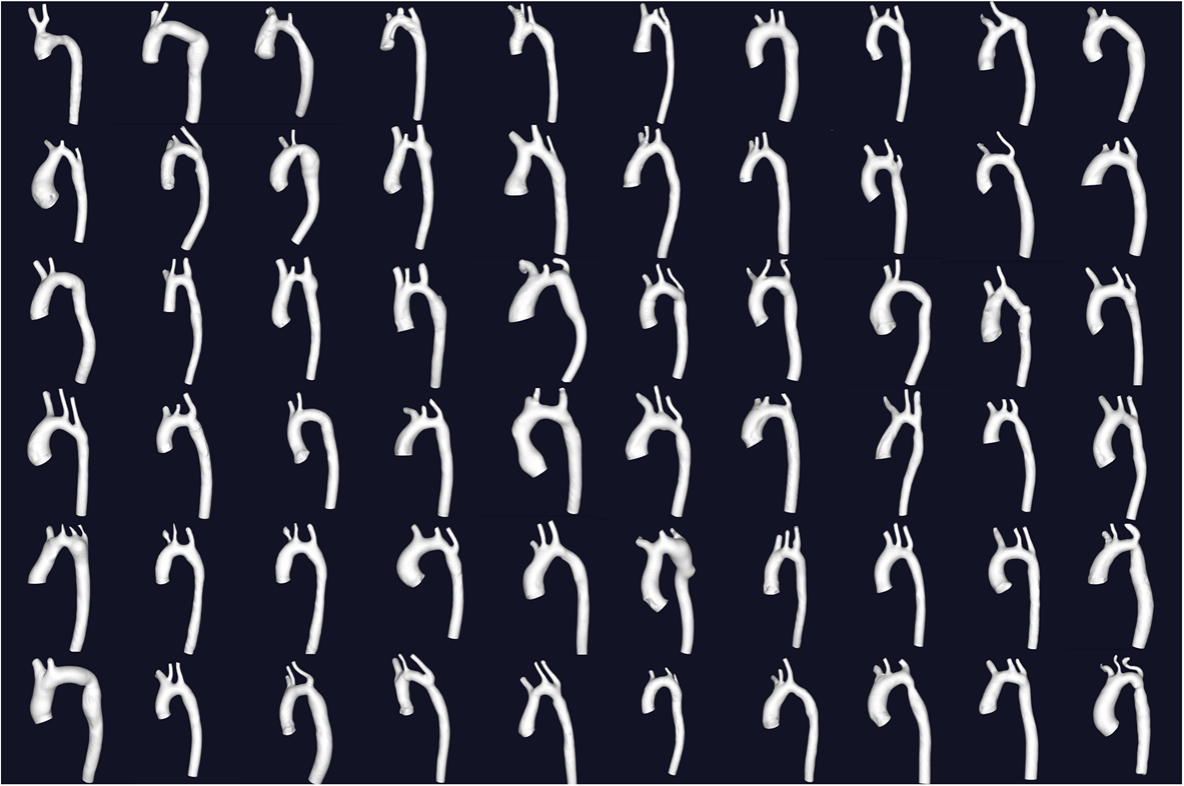
3D volume rendering images of the included aortas

The aorta was segmented from the CMR data using a level set segmentation with the deformable model initialized using colliding fronts (VMTKlab version 1.54, Orobix, Bergamo, Italy). The raw segmentations were smoothed using a non-shrinking surface smoothing algorithm [12] and then clipped at the levels of the sinotubular junction and diaphragm to create the final 3D volume (Fig. 1). The aortic centrelines (ignoring head and neck vessels) were created by first placing ‘seeds’ at the inlet and outlet of the aorta. The centreline was then calculated as the shortest path between these 2 points that was bounded by the Voronoi diagram of the vessel model. The resulting data consisted of the x, y, and z coordinates of between 80 and 155 points along the computed centreline. Each coordinate point was also associated with radius of the aorta at this position, determined by the radius of the maximum inscribed sphere.

Prior to PCA, all aortic centrelines were scaled to the same length (mean length in the population) and interpolated to 100 points. This scaled data was then rigidly registered (rotation and translation only) to a reference position (average best fit plane of all examples) to remove simple positional differences. The radius data was also scaled so that the mean radius along the aortic length was the same in all patients. After these manipulations, PCA was performed separately on the curvature (x,y,z coordinates) and scaled radius data (Matlab R2016B, Mathworks Inc., Natick, Massachusetts, USA). Principal component analysis is a statistical procedure by which data is projected onto a set of linearly uncorrelated (orthogonal) principal components. These principal components are ranked by the amount of variance they explain in the original data. In the context of this study, each component can be thought of as a proto-shape and individual patient radius and curvature data can be expressed as a weighted combination of these components. Thus, the weights for each patient express the importance of the various components in determining the geometry of that patient’s aorta. In this study, only the first 5 principal components were further considered.

The raw CMR angiographic data were also used to evaluate aortic arch anatomy by quantifying: i) arch index: the transverse arch diameter (between innominate and left common carotid artery) divided by the descending aorta diameter (at level of diaphragm), and ii) coarctation index: the aortic isthmus (repair site) diameter divided by the descending aorta diameter. All measurements were made by visualizing the short axis of the vessel using a multi-planar reformatting tool. The patient’s aortic arches were also subjectively identified as “gothic” if the arch had an acutely angulated conformation.

### Ascending aortic flow and area assessment

Ascending aortic (just above sino-tubuluar junction) flow and area data were acquired using a prospectively triggered, spiral, velocity encoded spoiled gradient echo sequence as previously described [11]. The flow-imaging plane was planned using orthogonal long axis cine images of the ascending aorta and was placed just above the sinotubular junction. This ensured that area data was minimally affected by through-plane motion of the aortic sinuses. These data were collected at high temporal resolution (9.6 ms) within a breath hold (11 s) allowing the data to be used for wave intensity analysis (WIA).

Aortic data were processed using an in-house plug-in for the open source DICOM software OsiriX (OsiriX Foundation, Geneva, Switzerland) [13]. Segmentation of the ascending aorta was performed on the modulus image using a previously validated semi-automatic registration-based algorithm [14]. The aortic region of interest (ROI) was manually adjusted as necessary to ensure optimal vessel wall delineation. The final ROIs were used to both calculate the aortic cross-sectional area and prescribe the region in the phase image from which flow and cardiac output were calculated.

### Derivation of central SBP using area-distension waveforms

Central aortic SBP (c-SBP) was calculated using a previously validated aortic area calibration method. Briefly, aortic areas waveforms were inputted into an exponential pressure-area model that was iteratively tuned to minimize the difference between the synthesised mean and DBP’s and the non-invasively measured pressures [1, 15]. This calibration scheme was based on the validated assumption that diastolic and mean blood pressures are conserved throughout the arterial system [16, 17]. The estimated c-SBP was then taken as the peak of the synthesised pressure curve and central pulse pressure (c-PP) was calculated as c-SBP-DBP.

### Total arterial compliance

Total arterial compliance (TAC) was calculated using a previously validated 2-element windkessel model [18, 19]. Briefly, the aortic flow curve was inputted into the Windkessel model with measured arterial resistance (MBP/CO) and TAC was tuned so that pulse pressure generated by the model equaled measured c-PP. The TAC index (TACi) was TAC divided by the body surface area (BSA).

### Wave intensity analysis (WIA)

In WIA, waves are regarded as a summation of incremental wave fronts; [20, 21] it is therefore possible to separate the flow (Q) and area (A) curves into the respective forward and backward components by expressing the relationship between wave speed and changes in flow and cross sectional area, as previously described [22]. Using this system Forward (Ejection) Compression Waves (FCW), and Backwards (reflected) Compression Waves (BCW) can be separated. The type of wave and their magnitude (area under the wave) were determined by analysis of the net and separated WIA plots. The areas under the separated waveforms were calculated by numerical integration.

### Left ventricular assessment

LV volumes and mass were assessed using a multi-slice real-time balanced steady state free precession sequence as previously described [23]. Images were acquired the LV short axis with full LV coverage (9–12 slices).

Quantification of end-diastolic and end-systolic volumes was performed by manual segmentation of the endocardial contour of short-axis cine images at end-diastole and end-systole using a built-in plug-in for OsiriX. From these LV ejection fraction (EF) was calculated and used as a marker of global systolic function. Epicardial contours were manually segmented at end systole. LV mass was calculated as the difference between the epicardial and endocardial contours multiplied by the slice thickness and a specific density of ventricular mass of 1.05 g/ml. The LV mass was divided by BSA to provide the indexed mass (LVMi), which was used as a marker of the cardiac response to abnormal load.

### 1D computer simulations

A validated 1d model of the systemic vascular tree [24] was used to explore role of the change in radius along the length of the aorta in generating wave reflections. This model solves the 1D Navier-Stokes equations and provides pressure and flow waveforms along the arterial tree. The model was run using a time-varying elastance model for the heart on its upstream boundary, such that simulated waves originate from the interaction of the pumping heart in the arterial tree. Separate models were created to represent the PC_rad_ that were associated with abnormal wave reflections in the clinical studies. Specifically, the radii of the aortic segments in the 1D model were set to equal corresponding radii of the ±2SD ‘proto-aorta’ for each selected PCs. Wave intensity analysis was then performed using conventional pressure and velocity methodology [21].

### Statistics

STATA (version 13.1, Stata Corporation, College Station, Texas, USA) was used for statistical analysis. Data were examined for normality and where appropriate, non-normally distributed variables were log transformed to ensure normal distribution prior to analysis. Descriptive statistics are expressed as mean (± standard deviation) when normally distributed, and geometric mean (± geometric standard deviation) when non-normally distributed, unless specified. Proportions are expressed as percentages.

Pearson’s correlation coefficient was used to analyse simple linear relationships between PCA, aortic length and wave intensity indices. Logistic regression was used to analyse the relationship between the subjective identification of a gothic arch and shape. Multivariable linear regression analysis was also used to determine the independence of associated covariates.

## Results

### Demographics and phenotype

The mean age of participants was 25.0 ± 7.8 years and 38 (63%) were male. Thirty-two (53%) patients had bicuspid aortic valves. The mean age at coarctation repair was 3.7 ± 12 months with the majority having an end-end anastomosis (72%). Eight (13%) patients had a history of aortic arch re-intervention after initial therapy: 6 catheter balloon angioplasties and 2 surgical re-interventions. Full description of timing and type of surgery, as well as additional procedures is included in supplemental materials (Additional file 1).

The mean p-SBP was 123 ± 14 mmHg and c-SBP was 115 ± 12 mmHg. The majority of patients (58%) had abnormal p-SBP: 5 patients (8%) were hypertensive (p-SBP > 140 mmHg). 16 patients (27%) were pre-hypertensive (p-SBP: 130-139 mmHg), 14 patients (23%) had ‘elevated BP’ (p-SBP: 120-129 mmHg). The remaining 25 patients (42%) were normotensive (p-SBP < 120 mmHg). Thirteen patients (22%) were receiving antihypertensive therapy at the time of assessment including 9 patients receiving monotherapy and 4 patients receiving two or more medications.

The TACi was 0.62 ± 0.14 ml/mmHg/m^2^. Mean BCW area was 0.0011 ± 0.0021 cm^5^ and FCW was 0.0092 ± 0.0012 cm^5^. The mean LVEF was 66 ± 7.5% and LVMi was 72 ± 14 g/m^2^.

### Aortic geometry

The mean coarctation index was 0.93 ± 0.55 and arch index was 1.05 ± 0.15. The mean end-diastolic ascending aortic diameter was 2.5 ± 0.48 cm (BSA indexed = 1.4 ± 0.24 cm/m^2^) and descending aortic dimeter was 1.8 ± 0.24 cm (BSA indexed = 1.0 ± 0.12 cm/m^2^). Fourteen patients (23%) were subjectively identified as having a gothic arch.

**Fig. 2:**
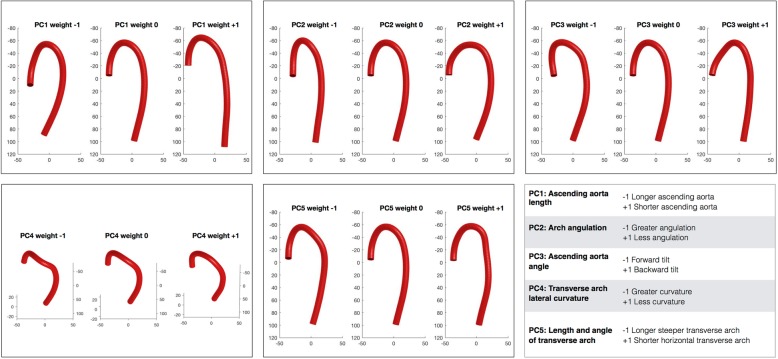
Principal component (PC) analysis (PCA) of 3D curvature, showing first 5 principal components (PC1–5). ‘Weight 0’ represents the mean aortic 3D curvature and Weight − 1 to + 1 represent ±2 standard deviations in each PC respectively

The first 5 principal components of aortic curvature (PC_curvature_) are shown in Fig. 2. They respectively account for 73, 11, 5, 4 and 3% of the variance in curvature in the population. The 1st PC_curvature_ primarily describes the relative length of the ascending aorta and the curve of the descending aorta. The 2nd PC_curvature_ describes the angulation of the arch and there was a significant association (*p* = 0.0036) between this component and subjective identification of a gothic arch. The 3rd describes the angle of the ascending aorta and the 4th describes the secondary curvature of the transverse arch. Finally, the 5th PC_curvature_ describes the length of the transverse arch and the angle of the proximal descending aorta.

**Fig. 3:**
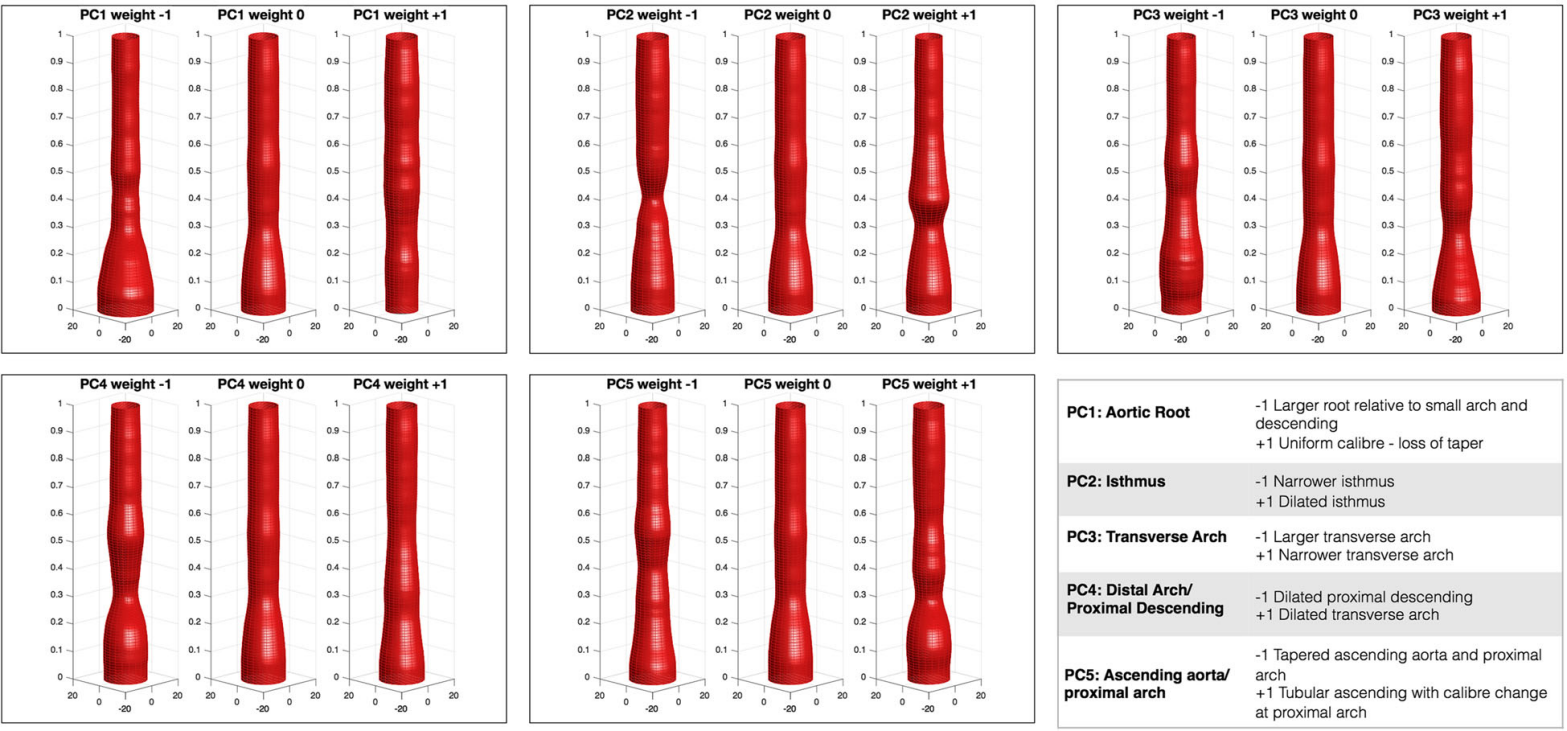
PCA of 3D radius, showing first 5 principal components. (PC1–5). ‘Weight 0’ represents the mean aortic 3D radius and weight − 1 to + 1 represent ±2 standard deviations in each PC respectively

The first 5 principal components of aortic radius (PC_radius_) are shown in Fig. 3. They respectively account for 48, 17, 10, 8, 6% of the variance in radius in the population. The 1st PC_radius_ primarily describes the size of the ascending aorta in relation to the rest of the aorta. The 2nd PC_radius_ describes the size of the isthmus relative to the descending aorta and was significantly associated with coarctation index (*r* = 0.51 *p* < 0.0001). The 3rd PC_radius_ describes the shape of the ascending aorta and relative size of the arch/proximal descending aorta. The 4th PC_radius_ describes size of the arch relative to the distal descending aorta and this component was significantly associated with arch index (r = 0.35, *p* = 0.007). The 5th PC_radius_ describes the shape of the ascending aorta and arch.

### Associations between curvature and radius components

There were significant associations between the 1st component of curvature and the 3rd and 4th components of radius (r = 0.31, *p* = 0.015; r = 0.29 *p* = 0.027 respectively). There was also a significant association between the 2nd component of curvature and 1st component of radius (r = − 0.29, *p* = 0.025). Finally, there was a significant association between the 3rd components of curvature and radius (r = 0.27, *p* = 0.035).

### Associations between geometry and hemodynamic

There was no association between subjective identification of a gothic arch and hemodynamic parameters (*p* > 0.2). Coarctation index was significantly associated with c-SBP (*r* = − 0.304, *p* = 0.018) and TACi (r = 0.269, *p* = 0.038). Arch index was significantly associated with c-SBP (r = − 0.256, *p* = 0.048).

The 1st, 3rd and 4th principal components of radius were associated with the area of the BCW (Table 1). Specifically, a large ascending aorta, transverse arch hypoplasia and a relatively smaller descending aorta all increase the area of the BCW. When these components were entered into a multiple regression model they were independently predictive of BCW area (r^2^ = 0.3, *p* < 0.02). The 3rd principal component was also associated with the area of the FCW (r = 0.28, *p* = 0.033) and 4th principal component with c-SBP (r = − 0.269, *p* = 0.037). No other significant association between the principal components of radius and hemodynamic parameters were found.

**Table 1:** Univariable linear relationships between shape indices and hemodynamic variables ^*^Log transformed for normality

	c-SBP		p-SBP		TACi*		BCW		FCW		LVMi		LVEF	
Variable	r	p	r	p	r	p	r	p	r	p	r	p	r	p
PC Curvature 1	− 0.100	0.448	− 0.146	0.267	0.019	0.887	−0.143	0.276	0.118	0.371	−0.006	0.966	0.106	0.420
PC Curvature 2	−0.189	0.149	−0.122	0.354	0.001	0.996	0.142	0.279	0.011	0.936	−0.057	0.665	−0.132	0.315
PC Curvature 3	0.039	0.765	0.066	0.617	−0.046	0.728	0.028	0.832	0.003	0.979	0.111	0.398	0.055	0.679
PC Curvature 4*	0.006	0.966	−0.146	0.267	0.178	0.174	0.025	0.848	−0.216	0.098	0.039	0.767	−0.176	0.178
PC Curvature 5	0.055	0.679	−0.048	0.715	0.119	0.367	0.222	0.088	0.173	0.185	0.050	0.703	−0.019	0.885
PC Radius 1	0.132	0.314	0.156	0.233	0.035	0.792	**−0.360**	**0.005**	0.072	0.585	0.143	0.275	−0.202	0.121
PC Radius 2*	−0.158	0.228	−0.170	0.195	0.226	0.082	−0.056	0.671	−0.085	0.519	0.023	0.860	0.094	0.476
PC Radius 3*	0.036	0.784	0.083	0.527	−0.075	0.567	**0.271**	**0.036**	**0.276**	**0.033**	−0.038	0.775	0.086	0.513
PC Radius 4	**−0.269**	**0.037**	−0.232	0.074	−0.087	0.511	**−0.306**	**0.017**	−0.075	0.571	−0.098	0.458	0.127	0.334
PC Radius 5	0.175	0.182	−0.020	0.878	−0.070	0.594	0.118	0.371	0.138	0.294	−0.043	0.744	−0.031	0.812
Coarctation index^a^	−0.234	0.073	**−0.304**	**0.018**	**0.269**	**0.038**	−0.054	0.682	−0.152	0.246	0.033	0.802	−0.038	0.776
Arch index	−0.242	0.062	**−0.256**	**0.048**	0.045	0.735	−0.175	0.182	−0.231	0.076	−0.089	0.499	0.043	0.742

Values in bold represent statistically significant associations

There were no significant associations (Table 1) between the principal components of curvature and i) blood pressure (c-SBP, c-PP, p-SBP): *p* > 0.12, ii) TACi, *p* > 0.2, iii) LV metrics (LVEF and LVMi): *p* > 0.15 and iv) results of wave intensity analysis (BCW and FCW): *p* > 0.82.

### In silico validation using 1D models

**Fig. 4:**
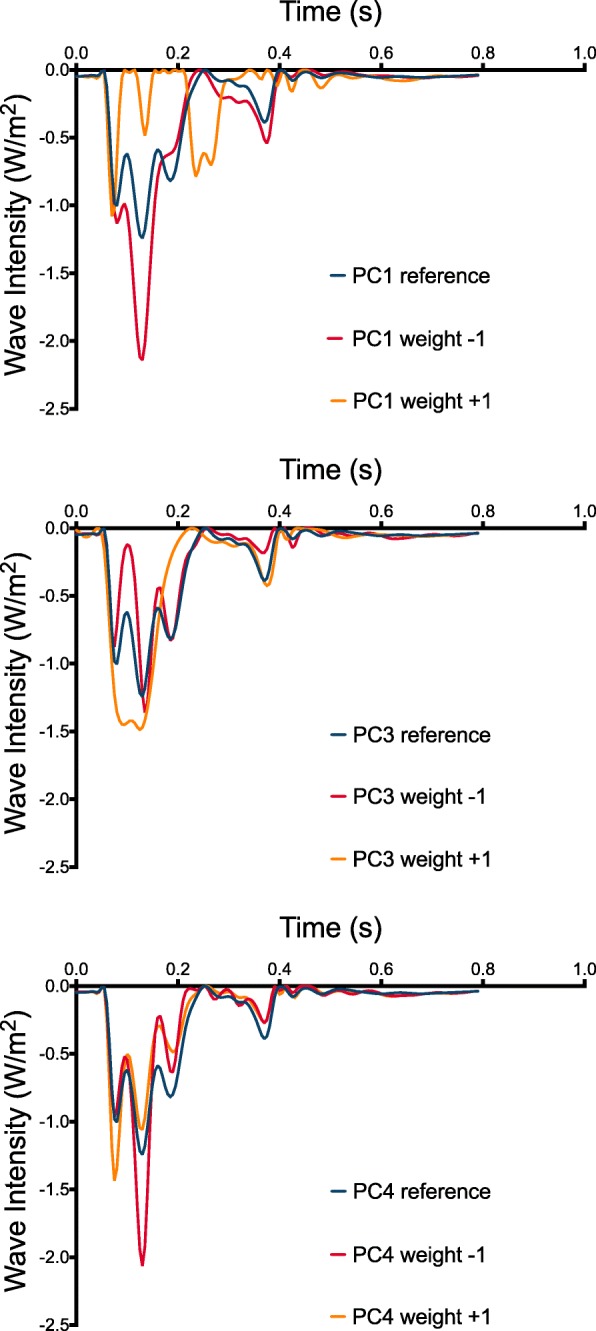
Patterns of negative wave intensity for 1D models based on the 1st, 3rd and 4th principle components of radius. For the model simulating the first PC of radius, the negative weight (red line) had an increased backwards compression wave (BCW) area, in keeping with the clinical data. The positive weight (yellow line) of the 3rd PC had a greater BCW area and the negative weight (red line) of the 4th PC had the largest BCW area. Both these findings were also in agreement with the clinical data

Aortas based upon ±2SD of the 1st, 3rd and 4th principle components of radius were selected for a 1D modelling study based on their statistical relationship with the BCW magnitude. Conventional wave intensity analysis for these models are shown in Fig. 4. All three models showed increased BCW area consistent with the patient data.

## Discussion

In this study, we independently quantified the separate components of aortic shape to fully investigate their role in determining hemodynamics in patients following coarctation repair. Our main findings include i) It is possible to use PCA to characterize the major modes of anatomical variation in arch anatomy, ii) There were no associations between curvature and either aortic hemodynamics or LV mass or function, iii) changes in radius along the aortic length had a modest association with the magnitude of the BCW, and iv) 1D haemodynamic models of aortic shapes produced patterns of wave reflection consistent with clinical findings. These data suggest that aortic shape has only a moderate influence on abnormal vascular load after coarctation repair.

Abnormal aortic shape is often implicated as a significant mediator of increased load after coarctation repair [4, 5]. However, evaluation of aortic shape is often qualitative (i.e. identification of a gothic arch) or overly simplistic (i.e. simple height/width ratios). This may be why several studies have produced contradictory results regarding the link between shape and hemodynamic parameters [6]. Therefore, in this study we sought to accurately quantify shape and, importantly, separately investigate the 2 aspects of shape: radius and curvature. To accomplish this, we used PCA, which is a well-recognised method of assessing shape [25]. PCA can be applied to any collection of data points and extracts the components of that data that explain the greatest variance. When applied to aortas, these components can be thought of as the shapes that explain the greatest variation in the study population. Importantly, individual patient aortas can be represented as the combination of differing proportions of these components. Thus, PCA allows quantification of aortic shape that is based on statistical variation rather than simple geometric assumptions.

In the literature, curvature and ‘shape’ are often conflated with most definitions of abnormal shape (i.e. the gothic arch) actually relating to curvature. We have shown that curvature was not associated with any of the global aortic hemodynamic parameters. Importantly, our analysis included backwards wave reflections, which are often implicated as the mediator of increased load in ‘gothic arches’. Of course, it is possible that our PCA methodology was not well suited to extracting the aspects of curvature that result in abnormal hemodynamics. However, the extracted principal components of curvature do seem to reflect the common morphologies seen in patients after coarctation repair. Furthermore, the 2nd component of curvature was associated with subjective identification of a gothic arch. Thus, we believe that our analysis does provide an accurate quantification of curvature and the lack of association with hemodynamics is a real finding. This is corroborated by the fact that there is no association between curvature and LVEF and LVMI.

The fact that curvature does not significantly influence wave reflections is not surprising from a mechanical point of view. The wave lengths of pressure and flow waves are in the order of meter, much larger than the geometrical dimensions of the aorta and bends and curves, explaining why arterial wave dynamics are very well described using 1D formulations of the momentum equations [26].

An aspect of this study, which is unusual is that we separately investigated the change in radius along the aortic length. This is an aspect of shape that is either neglected, simplified down to single indices (i.e. arch and coarctation index) [1] or combined with curvature [8]. We have shown that several principal components of radius were associated with increased BCW. Specifically, a large ascending aorta narrowing to a relatively smaller arch and descending, or transverse arch hypoplasia resulted in significantly increased BCW’s. This is in keeping with radius changes causing impedance mismatches that result in reflections. However, it should be noted that we did not adjust for multiple correlation tests. This was done to ensure that true associations were not rejected, but increased the risk of false discoveries. Thus, we also corroborated our clinical findings with 1D in-silico models based on clinical radius data. The fact that these models also exhibited increased BCW’s suggests that the relationship between change in radius and wave reflection is true. It should be noted that the radius components only account for approximately 30% of the variance in BCW. Thus, other factors must come in to play, the most important probably being abnormal aortic stiffness, particularly at the repair site [27].

A question that remains to be answered is why studies have shown a relationship between shape (i.e. gothic arch) and hemodynamics [4]. One possibility raised by this study is that certain curvatures are associated with radius changes that predispose to increased reflections. For instance, the 2nd component of curvature (linked to our identification of a gothic arch) is associated with the 1st component of radius, which is one of the main predictors of wave reflections. Other curvature components that have a similarity to the gothic arch (1st and 3rd) are also associated with radius components that are associated with greater reflections. Thus, the findings of previous studies that have connected to the curvature aspect of shape to hemodynamics may simply be due to the radius aspect of shape. Clinically, this means we should be more concerned with radius change rather than with simple curvature-based description of shape such as the gothic arch.

### Limitations

A limitation of our study is that only a minority of patients were truly hypertensive. However, the majority (58%) did have some elevation of SBP. It is recognized that even small increases in blood pressure add to cumulative cardiovascular risk.[28]We were limited also limited by the lack of ambulatory or exercise blood pressure data. It is possible that during exercise a combination of abnormal curvature and increased cardiac output might result in an excessive increase in blood pressure. However, if this were the case, one might expect an association between curvature and LV mass as this is integrates load over all normal activities. The lack of such an association implies that curvature is not an important mediator of exercise load.

Another limitation is the use of PCA to determine the major modes of shape variation. PCA is an unsupervised method, which extracts the major modes of variation based on the population alone. In our study, this might limit the ability to find rarer shape variants that are associated with hemodynamic parameters. Alternatively, supervised methods (i.e. partial least squares regression) that inherently include outcome variables could be used. Previously, partial least squares regression has been used to associate aortic shape to hemodynamic parameters [7, 8]. However, this type of approach is prone to overfitting and identification of shape variants that may not be prevalent in the population. Thus, we believe that our approach is more robust as it reflects the major and thus more important variations in the population studied.

A final limitation is that we have not assessed the possible effect of radius and curvature changes on flow patterns in the aorta. In particular, it is possible that abnormal shape may affect flow patterns in such a way to reduce hemodynamic efficiency. In the future, this could be investigated by using 4D flow to evaluate helical flow patterns and measure turbulence.

## Conclusions

We demonstrate the major modes of variation in 3D aortic shape in patients post coarctation repair. We observed a modest association between variation in 3D radius and pathological wave reflections but no association with 3D curvature and any hemodynamic parameters. These data suggest that shape is not a major determinant of vascular load following coarctation repair and that variation in vessel calibre is more important than curvature.

## Additional file

Additional file 1: Supplemental Demographics. (DOCX 12 kb)

## Abbreviations

A: Area
BCW: Backwards compression waves
BSA: Body surface area
CMR: Cardiovascular magnetic resonance
c-PP: Central pulse pressure
c-SBP: Central systolic blood pressure
DBP: Diastolic blood pressure
EF: Ejection fraction
FCW: Forward compression waves
LV: Left ventricle/left ventricular
LVMi: Left ventricular mass index
MBP: Mean blood pressure
PCA: Principal component analysis
Q: Flow
ROI: Region of interest
SBP: Systolic blood pressure
TAC: Total arterial compliance
TACi: Indexed total arterial compliance
WIA: Wave intensity analysis
