# Correction to: The aorta after coarctation repair – effects of calibre and curvature on arterial haemodynamics

**DOI:** 10.1186/s12968-019-0540-9

**Published:** 2019-05-23

**Authors:** Michael A. Quail, Patrick Segers, Jennifer A. Steeden, Vivek Muthurangu

**Affiliations:** 1 Centre for Translational Cardiovascular Imaging, Institute of Cardiovascular Science, University College London and Great Ormond Street Hospital for Children, London, WC1N 3JH UK; 2 IBiTech-bioMMeda, iMinds Medical IT, Ghent University, De Pintelaan 185, 9000 Ghent, Belgium

**Correction to: J Cardiovasc Magn Reson**

**https://doi.org/10.1186/s12968-019-0534-7**

In the original version of this article [1], published on 11 April 2019, there is 1 error in the ‘Conclusion’ paragraph of the abstract.

The wording of the abstract in the original publication is as followed

- There are major modes of variation in 3D aortic shape after coarctation repair with a modest association between variation in aortic radius and pathological wave reflections, but not with 3D curvature.

The corrected information is as followed:

- We have demonstrated the major modes of variation in 3D aortic shape after coarctation repair. There exists a modest association between variation in aortic radius and pathological wave reflections but not with 3D curvature.
