# Supplementary material for: The aorta after coarctation repair – effects of calibre and curvature on arterial haemodynamics
Source: J Cardiovasc Magn Reson. 2019 Apr 11;21:22. doi: 10.1186/s12968-019-0534-7 (PMC6458643; doi:10.1186/s12968-019-0534-7)
Supplement: Supplementary file 1 — Supplemental Demographics. (DOCX 12 kb) [file 12968_2019_534_MOESM1_ESM.docx]

Supplemental Demographics

| Age of Repair  Neonatal Repair  Repair <1year  Childhood <16years  Adulthood >16 years  Unknown Age | 22 (37%)  17 (28%)  17 (28%)  2 (3%)  2 (3%) |
| --- | --- |
| Type of Repair  End-to-End Anastomosis  Extended End-to-End Anastomosis  Subclavian Flap Angioplasty  Dacron/Gore-Tex Angioplasty | 43 (72%)  5 (8%)  9 (15%)  3 (5%) |
| Secondary Re-intervention  Balloon Angioplasty  Surgical Angioplasty | 6 (9%)  2 (3%) |
| Additional Cardiac Lesions  Bicuspid Aortic Valve  Ventricular Septal Defect  (resolved/repaired)  Supramitral membrane  Cor triatriatum  Repaired subaortic stenosis | 26 (52%)  21 (35%)  1 (2%)  1 (2%)  1 (2%) |
